# The Fabrication and Evaluation of a Potential Biomaterial Produced with Stem Cell Sheet Technology for Future Regenerative Medicine

**DOI:** 10.1155/2020/9567362

**Published:** 2020-02-10

**Authors:** Shukui Zhou, Ying Wang, Kaile Zhang, Nailong Cao, Ranxing Yang, Jianwen Huang, Weixin Zhao, Mahbubur Rahman, Hong Liao, Qiang Fu

**Affiliations:** ^1^Department of Urology, Sichuan Cancer Hospital & Institute, Sichuan Cancer Center, School of Medicine, University of Electronic Science and Technology of China, Chengdu, China; ^2^Department of Urology, Affiliated Sixth People's Hospital, Shanghai Jiao Tong University, Shanghai, China; ^3^Wake Forest Institute for Regenerative Medicine, Winston Salem, NC, USA; ^4^Department of General Educational Development (GED), Faculty of Science & Information Technology, Daffodil International University, Dhaka, Bangladesh

## Abstract

To date, the decellularized scaffold has been widely explored as a source of biological scaffolds for regenerative medicine. However, the acellular matrix derived from natural tissues and organs has a lot of defects, including the limited amount of autogenous tissue and surgical complication such as risk of blood loss, wound infection, pain, shock, and functional damage in the donor part of the body. In this study, we prepared acellular matrix using adipose-derived stem cell (ADSC) sheets and evaluate the cellular compatibility and immunoreactivity. The ADSC sheets were fabricated and subsequently decellularized using repeated freeze-thaw, Triton X-100 and SDS decellularization. Oral mucosal epithelial cells were seeded onto the decellularized ADSC sheets to evaluate the cell replantation ability, and silk fibroin was used as the control. Then, acellular matrix was transplanted onto subcutaneous tissue for 1 week or 3 weeks; H&E staining and immunohistochemical analysis of CD68 expression and quantitative real-time PCR (qPCR) were performed to evaluate the immunogenicity and biocompatibility. The ADSC sheet-derived ECM scaffolds preserved the three-dimensional architecture of ECM and retained the cytokines by Triton X-100 decellularization protocols. Compared with silk fibroin in vitro, the oral mucosal epithelial cells survived better on the decellularized ADSC sheets with an intact and consecutive epidermal cellular layer. Compared with porcine small intestinal submucosa (SIS) in vivo, the homogeneous decellularized ADSC sheets had less monocyte-macrophage infiltrating in vivo implantation. During 3 weeks after transplantation, the mRNA expression of cytokines, such as IL-4/IL-10, was obviously higher in decellularized ADSC sheets than that of porcine SIS. A Triton X-100 method can achieve effective cell removal, retain major ECM components, and preserve the ultrastructure of ADSC sheets. The decellularized ADSC sheets possess good recellularization capacity and excellent biocompatibility. This study demonstrated the potential suitability of utilizing acellular matrix from ADSC sheets for soft tissue regeneration and repair.

## 1. Introduction

To date, the decellularized scaffold has been widely explored as a source of biological scaffolds for regenerative medicine and tissue engineering. Compared with artificial synthetic biomaterials, the decellularized scaffold obtains the nature-designed architecture, retains the inherent growth factor to promote cellular growth, and restores the organ function [1]. Many studies have focused on the decellularization of natural tissues and organs, including the blood vessel [2], skin [3], small intestinal submucosa [4], urinary bladder [5], adipose tissue [6], spleen [7], and lung [8]. Their shortcomings include the limited amount of autogenous tissue derived from the patient, increased operation time, postoperative recovery time, and surgical complication such as risk of blood loss, wound infection, pain, shock, and functional damage in the donor part of the body [9]. Furthermore, current decellularization techniques are not able to remove the cellular components completely. Xenogeneic cell remnants within the decellularized scaffolds may lead to adverse host immune responses *in vivo*. Thus, there is a need to explore alternative solutions.

Cell sheet technology facilitated us to harvest confluent cells for fabricating a contiguous cell sheet with intact extracellular matrix (ECM). ECM is an ideal biological material for tissue engineering and regenerative medicine, which provides a structural and nutritive microenvironment for cell differentiation and proliferation and avoids the disadvantages of using exogenous scaffold, such as toxicity, inflammatory response, and uneven cell distribution [10]. The time and thickness of cell sheet formation were dependent on the capability of cell proliferation. Adipose-derived stem cells (ADSCs) are the most commonly used stem cell types in autoplastic transplantation. On the one hand, compared with mesenchymal stem cells derived from bone marrow and cartilage, the ADSCs possess the highest proliferation potential and exhibit high tolerance to serum deprivation-induced apoptosis [11]. On the other hand, adipose tissue is abundant in the body and contains a high content of ADSCs; approximately 0.7 × 10^6^ ADSCs can be isolated in a gram of adipose tissue [12]. Moreover, adipose tissue can be easily obtained in large quantities with little donor site morbidity or patient discomfort. What is more, mesenchymal stromal cells are proved to be the robust source of chemokines and cytokines, which promote the growth, differentiation, and migration of cells and lead to fast recellularization of the biomaterials [13].

In recent years, ADSC sheet transplantation has shown the potential to be used widely for repair and reconstruction of damaged tissues and organs, including myocardial infarction [14], diabetic ulcers [15], and full-thickness defect wound [16]. Since the cell sheet is composed of cells and compact ECM, thus, the decellularized cell sheet has the potential to be used as a novel biological scaffold for regenerative medicine. Cell-derived ECM overcomes the issues of possible exogenous pathogen transferring and allows ECM produced by the patients' own cells [17]. Due to the prevalence of liposuction surgeries, adipose tissue is uniquely accessible from living human donors and the decellularized ADSC sheets have a good prospect of clinical application without the ethical issues. In this study, we investigated the effectiveness of three typical methods for ADSC sheet decellularization and assess the ECM structure, growth factor retention, recellularization potential, and histocompatibility of the decellularized ADSC sheets.

## 2. Materials and Methods

### 2.1. Materials

All the chemicals used for ADSC sheet decellularization were obtained from Sigma-Aldrich unless otherwise specified. The cell culture reagents and products, such as the 60 mm temperature-responsive cell culture dishes, were purchased from Thermo Fisher Scientific (Rockford, IL, USA). Beagle dogs at 10 months of age were provided by the animal laboratory of Shanghai Sixth People's Hospital. The animal experiments were reviewed and approved by the Animal Experimental Ethics Committee of the hospital according to the guidelines for the ethical treatment of animals established by the international council for laboratory animal science.

### 2.2. Primary Cell Isolation and Culture

As a common large experimental animal, canine was used in our study for abundant subcutaneous fat, similar to human physiology and strong disease resistance. ADSCs were obtained by the procedure previously reported and briefly isolated by enzymatic digestion [18]. Fat tissue was collected from the groin area of beagle dogs. The tissue sample was washed three times with 0.25% Chloromycetin solution and phosphate-buffered saline buffer (PBS) and then digested with 0.1% collagenase I for 1 hour at 37°C. The dissociated cells were collected after filtration through a cell strainer with the pore diameter of 40 *μ*m. The primary ADSCs were plated onto 100 mm dishes at 37°C and a humidified atmosphere with 5% CO_2_ and maintained with low glucose Dulbecco's modified Eagle's medium (DMEM; Gibco, NY, USA) supplemented with 10% fetal bovine serum (FBS; Gibco), 100 mg/mL streptomycin sulfate (Gibco), and 100 U/mL penicillin (Gibco). The ADSCs were used at passage 3 in the subsequent experiments, and the hematopoietic marker CD45 and the stem cell markers CD105 and CD90 were evaluated by flow cytometry.

Oral mucosal epithelial cells were harvested from canine oral mucosal epithelium and prepared as reported previously [19]. Oral mucosal epithelial cells were obtained from the buccal mucosa of beagle dogs. The harvested 1.5 cm × 1.5 cm oral buccal mucosa tissue was washed 3 times with 0.25% chloramphenicol solution, cut into small pieces, and digested overnight at 4°C with 0.25% neutral protease (Dispase-II, Sigma-Aldrich). Then, the epithelial layer was separated with tweezers and digested in 0.05% pancreatic enzymes (Gibco) for 10 min. The suspended cells were filtered, centrifuged, and cultured in keratinocyte serum-free medium (K-SFM, Gibco) supplemented with 5 *μ*g/L human recombinant epidermal growth factor and 50 *μ*g/mL bovine pituitary extract. Primary cells at passage 2 were used in further experiments. The cell culture medium was changed at 2-day intervals.

### 2.3. Formation of ADSC Sheets

The vigorous ADSCs were harvested for further experiments. To create cell sheets, ADSCs at 5 × 10^4^/cm^2^ were seeded in a 60 mm temperature-responsive cell culture surface (Thermo Fisher Scientific, San Jose, CA, USA). The culture medium was composed of low-glucose DMEM, 10% FBS, 100 mg/mL streptomycin sulfate, and 100 U/mL penicillin. Once reaching about 80%-90% confluence, the ADSCs were stimulated with 100 *μ*g/mL vitamin C (Sigma-Aldrich, St. Louis, MO) to stimulate extracellular matrix deposition. The culture medium was replaced every 2 days. When canine ADSCs were cultured for 21 days at 37°C in 5% CO_2_, we reduced the culture temperature to 20°C for 30 minute to obtain intact cell sheets.

### 2.4. Decellularization of ADSC Sheets

During the process of cell lysis caused by decellularized treatment, many kinds of intracellular proteases are released which may cause undesirable damage to the native ECM [20]. To protect the ECM from these proteases, serine protease inhibitors, such as aprotinin (Sigma-Aldrich, USA), are used to protect ECM and intracellular protease interactions. The decellularization treatments were divided into three groups: (1) Freeze-thaw method: freeze-thaw step at -80°C for 2 h and subsequently thawed in phosphate-buffered saline (PBS) solution containing 1 *μ*g/mL aprotinin at room temperature (25°C) for 30 min and rinsed in PBS for 24 h; this process was repeated three times. (2) Triton X-100 method: the ADSC sheets were placed at the decellularization solution, containing 1% Triton X-100, 0.02% EDTA, 10 mM Tris, and 1 *μ*g/mL aprotinin, shaking the ADSC sheets for 24 h at room temperature, and rinsed in phosphate-buffered saline (PBS) for 24 h. (3) SDS method: the ADSC sheets were placed in the decellularization solution containing 0.25% sodium dodecyl sulfate (SDS), 0.02% EDTA, 10 mM Tris, and 1 *μ*g/mL aprotinin; then the ADSC sheets were oscillated for 2 h at room temperature and washed with PBS for 24 h to remove residual reagents. The ADSC sheets without decellularization were set as the control.

### 2.5. DNA Quantification

All the samples were digested using proteinase K solution for 2 h at 37°C. Digested samples were centrifuged at 18,000g for 10 min at room temperature. The supernatant of each group was submitted to the PicoGreen DNA assay (Invitrogen) and adjusted for dry weight and normalized for blank samples. Total DNA concentration was measured with a spectrophotometer (Techno Scientific, CELBIO, Milan, Italy) by reading the optical density (OD) at *γ* = 260 nm, corresponding to the maximum absorption of nitrogenous bases.

### 2.6. Observation of the Residual Cell Components

The decellularization efficiency of the cell sheet was observed by scanning electron microscopy (SEM) and hematoxylin and eosin (H&E) staining. H&E staining was used to assess the removal of cellular components, and SEM was used to observe morphological change before and after the process of decellularization. For histological analysis, decellularized and native ADSC sheets were fixed for 2 h in 4% paraformaldehyde solution, dehydrated with a graded ethanol series, and embedded in paraffin. Then, 5 *μ*m thick sections were obtained by means of a microtome and stained with H&E staining. For SEM, samples were fixed with 2.5% glutaraldehyde in PBS for 4 hours. After thoroughly washing with PBS, the cells were gradually dehydrated and then dried by lyophilization. The specimens were then sputter coated with platinum and examined with a scanning electron microscope (SU8000 series; HITACHI, Tokyo, Japan).

### 2.7. Analysis of Retained Cytokines

ELISA analysis was used to determine the retained cytokines in the ECM. Soluble molecules were extracted from the ADSC sheets before and after decellularization with protein extraction kit (Invitrogen, Carlsbad, CA) and a protease inhibitor cocktail (Roche Applied Science, Indianapolis, IN). Then the samples were homogenized. The extracted lysates were centrifuged at 15,000 RPM for 10 min, and the supernatant was collected. According to the manufacturer's instructions, ELISA assays (Kaiji Bioengineering, Nanjing, China) were performed to determine transforming growth factor beta (TGF-*β*), basic fibroblast growth factor (bFGF), and vascular endothelial growth factor (VEGF) levels in the extracted lysates of each group.

### 2.8. Mechanical Testing

Mechanical tests were used to measure the elasticity of ADSC sheets before and after decellularization. The cutting size of cell sheets in each group was about 30 × 25 mm. The gripping distance is set to 10 mm. The load-displacement curve was carried out on longitudinally cut sheets by a micro Materials Test System (MTF-100, Shanghai University, China) at 10 mm/min until rupture occurred. The tension and the displacement are sampled 10 times per second, and the samples were kept wet with PBS during the test.

### 2.9. Cell-Seeding Potential

Silk fibroin was reported to be a potential biomaterial for regenerative medicine and has potential for use in the urethral reconstruction [21]. Silk fibroin was provided by the State Key Laboratory for Modification of Chemical Fibers and Polymer Materials, from the College of Materials Science and Engineering of Donghua University, Shanghai, China. In this study, we observed the cell-seeding potential of decellularized ADSC sheets compared with silk fibroin. The decellularized ADSC sheets were immersed in high-glucose DMEM containing 10% FBS for 2 h in a cell culture incubator (37°C). The oral mucosal epithelial cells were trypsinized and seeded onto the silk fibroin and decellularized ADSC sheets at a density of 0.5 × 10^4^ cells/cm^2^ in a 6-well plate, respectively. The cell-loaded constructs were incubated for 4 h before the supplemented culture medium was slowly added. The culture medium was changed every 2 days. Samples were observed with SEM on days 3 and 7 after cell seeding. Meanwhile, after 3 days and 7 days of cell culture, the cell-loaded constructs were fixed in 4% paraformaldehyde for 12 h and embedded in paraffin. Then, 5 mm serial sections were generated and evaluated by H&E staining.

### 2.10. Cell Proliferation Assay

The cell counting kit-8 (CCK-8; KeyGen Biotech, Nanjing, China) was used to evaluate the growth kinetics of oral mucosal epithelial cells on the scaffolds of decellularized ADSC sheets and silk fibroin. The decellularized ADSC sheets and silk fibroin were placed in 96-well microplates. A total of 2 × 10^3^/well oral mucosal epithelial cells were seeded on the decellularized ADSC sheets and silk fibroin in a total volume of 100 *μ*L, respectively. CCK-8 solution (10 *μ*L) was added to each well and incubated at 37°C for 30 min. Subsequently, the absorbance values of each well were measured by a microplate reader at 450 nm on days 1, 3, 5, and 7 after cell seeding. The absorbance values at different time points were used to construct a growth curve to contrast the cell proliferative ability of the two scaffolds.

### 2.11. Biocompatibility

Commercial small intestinal submucosa (SIS) (Cook Urological, Spencer, IN, USA) is the most extensively clinically used ECM and is derived from the small intestinal mucosa tissue of the pigs. Male Sprague–Dawley rats, aging 6-8 weeks, were purchased from Shanghai Slack Laboratory Animal Co. Ltd. (Shanghai, China). The rats were anesthetized with pentobarbital sodium (30 mg/kg) by intraperitoneal injection. A groin skin incision of 1.5 cm was made; then the area of 1 × 1 cm^2^ decellularized ADSC sheets or SIS was subcutaneously implanted in the rat's groin (xenoplastic transplantation) with a cell lifter to study the biocompatibility in vivo, and the tissue samples were retrieved at 1 week and 3 weeks. To evaluate the host-graft inflammatory reaction, H&E staining was performed to observe mononuclear cells and multinucleated foreign-body giant cell infiltration in the transplantation site. Immunohistochemical analysis was performed using CD68 (1 : 800 dilution, Abcam). The slides were washed with Tris/HCl-buffered saline (TBS) plus 0.025% Triton X-100 with gentle agitation and then blocked in TBS containing 5% bovine serum albumin for 2 hours at room temperature. The samples were treated with CD68 primary antibodies and incubated overnight at 4°C, followed by washing 5 times with PBS. The sections were then incubated for 1 hour at room temperature with secondary antibody (1 : 1500), followed by subsequent linking to horseradish peroxidase and substrate/chromogen reaction using immunoperoxidase secondary detection kit (Millipore, Billerica, MA). Th1 cells, which secrete IL-2 and IFN-*γ*, trigger phagocyte-dependent inflammation and cell-mediated immunity. Th2 cells, which produce IL-4 and IL-10, evoke humoral immunity (antibody production) and eosinophil accumulation [22]. Quantitative real-time PCR (qPCR) was used to measure the mRNA levels of local cytokine, including IL-2, IFN-*γ*, IL-4, and IL-10, and assess the immunoreactivity and immunogenicity of acellular tissues. The rat groin normal subcutaneous tissue was set as the control group. Total RNA of retrieved tissue (SIS and decellularized ADSC sheets) was extracted with Trizol (Invitrogen, Carlsbad, CA) and then reverse transcribed into cDNA with a quantitative RT-PCR Kit (TaKaRa; Shiga, Japan). The PCR analysis of target genes was performed in StepOnePlus™ Real-Time PCR Systems (Life Technologies, Singapore) using SYBR green PCR Master Mix Kit (Life Technologies). Primer sequences were designed and purchased from Shanghai Sangon Company (Shanghai, China). PCR was performed with primers: IL-2 (forward 5′-TTGTCTTGCATCGCACTGAC-3′, reverse 5′-GAGAGGTCCTACGAGTGTAA-3′), IFN-*γ* (forward 5′-CACCCGAACCTCTTCCTT-3′, reverse 5′-TCCCTGGTTCATCCGTCGGTT-3′), IL-4 (forward 5′-TCCCAACTGATTCCAACTCTG-3′, reverse 5′-CTTGTAGGAGTGTCGCTCTT-3′), and IL-10 (forward 5′-GAGTCGAGAAGAGTTGCCATC-3′, reverse 5′-CTACCGTTGAGAAGAGCTGAG-3′). The dog GAPDH was chosen as the reference gene (forward 5′-TAACTCTGGCAAAGTGGATATT-3′, reverse 5′-ATGACAAGTTTCCCGTTCTC-3′). PCR conditions are as follows: 35 cycles of amplification with 30 seconds of denaturation at 95°C, 30 seconds of annealing at 58°C, and 30 seconds of extension at 72°C with a final elongation step of 5 minutes at 72°C. Fold variation in gene expression was quantified using the comparative Ct method: 2^(CtTreatment − CtControl)^.

### 2.12. Statistical Analysis

Data were expressed as the mean ± standard deviation. Significant differences between groups were estimated using Student's *t*-test and/or nonparametric test. Statistical analysis was performed with SPSS 17.0 (IBM, NY, USA). *P* values of less than 0.05 were considered significant.

## 3. Results

### 3.1. ADSC Culture and ADSC Sheet Formation

**Figure 1:**
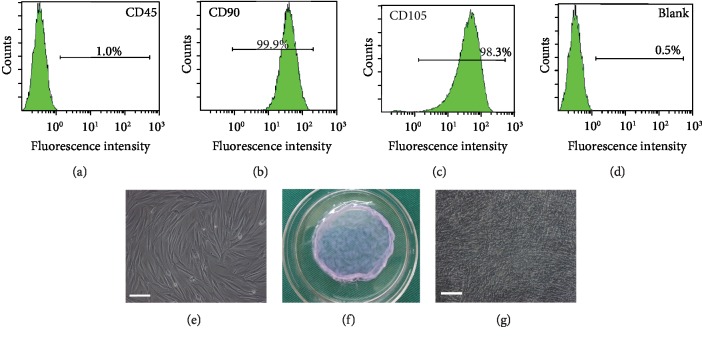
Canine ADSC culture and ADSC sheet formation. (a–d) ADSCs were negative for hematopoietic marker CD45 and were strongly positive for MSC-related markers CD90 and CD105. The unlabeled cells were used as the blank control. (e) The primary cultured ADSCs were isolated from fat tissue of beagle dogs (scale bars: 100 *μ*m). (f) ADSC sheets were obtained after 21 days of continuous cell culture. (g) ADSC sheets observed by inverted phase contrast microscopy (×100) and cell tight junction and surrounded by abundant ECM (scale bars: 100 *μ*m).

The primary cultured ADSCs adhered to the plate proliferate rapidly in vitro. ADSCs can be identified by the combination of stem cell-specific surface markers. ADSCs can express several detectable cell-specific proteins and CD markers, such as the positive protein markers, including CD29, CD44, CD73,CD90, CD105, and CD166, and lack the expression of the hematopoietic markers CD45 and CD34 [23]. Flow cytometry analysis indicated that the primary cultured ADSCs in our study were negative for hematopoietic marker CD45 and were strongly positive for MSC-related markers CD90 and CD105 (Figures 1(a)–1(d)), which confirmed the stem cell origin of ADSCs. ADSCs appeared to be centrally spirally distributed and are mostly seen in a long fusiform shape with single nuclei (Figure 1(e)). Canine ADSCs were cultured continuously for 21 days, and ADSC sheets were obtained by reducing the temperature method to 25°C for 30 min (Figure 1(f)). Under inverted phase contrast microscopy, the cells intermingled with each other closely and surrounded by abundant ECM (Figure 1(g)).

### 3.2. Evaluation of Decellularized Efficiency with Different Methods

**Figure 2:**
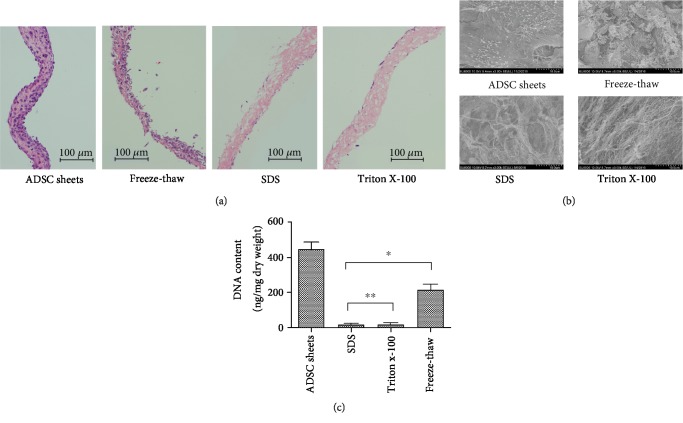
Evaluation of decellularized efficiency with repeated freeze-thaw, Triton X-100, and SDS decellularization. (a) H&E staining after the decellularization treatment. Compared with freeze-thaw treatments, SDS and Triton X-100 treatments removed the cellular contents more efficiently. The repeated freeze-thaw method with cell components residual, SDS method with disturbed and partial rupture collagen fiber, and decellularized Triton X-100 sample with an intact preserved collagen fiber structure. (b) Microstructural observations by scanning electron microscopy. (c) DNA quantification of different groups. Compared with the repeated freeze-thaw method, the methods of Triton X-100 and SDS more effectively reduced the DNA content (^∗^*P* < 0.05), but no statistically significant differences were found between these two groups (^∗∗^*P* > 0.05).

H&E staining before and after the decellularization treatment was used to assess efficient removal of cell components and preservation of the ECM architecture (Figure 2(a)). The H&E staining showed that ADSC sheets were composed of 5-7 layers of cells with a mean thickness of 74 ± 8.1 *μ*m. Compared with the repeated freeze-thaw method, cells could be effectively removed by the methods of SDS and Triton X-100. Triton X-100 samples retained the arrangements of collagen, while the original ECM structure has been destroyed in the SDS decellularized samples and the mesh of collagen fibers was disturbed, even partially ruptured. SEM images revealed an intense and uniform ECM microstructure in natural ADSC sheets (Figure 2(b)). The protein fibers were clearly exposed when adhesive molecules and proteins were removed in the decellularization processes. Massive cell components retained in the freeze-thaw-treated sample. The SDS decellularization procedures had a high clearance rate of the cellular components under observation of H&E staining and SEM images, but it also induced collagen fiber structural damage of the scaffold, appearing as dissociate and disrupted proteins in the ECM. In the decellularized Triton X-100 sample, collagen fibers can be identified and appear to be well-organized, without any cell remnants or cellular debris. Then, to evaluate the efficacy of removal of all cellular constituents from cell sheet, we performed DNA quantification analysis of three decellularization treatments. As shown in Figure 2(c), compared with the repeated freeze-thaw method, the methods of Triton X-100 and SDS reduced the DNA content more effectively (*P* < 0.05). The amount of DNA in the ADSC sheets after decellularization was 216 ± 27, 19 ± 6, and 17 ± 4 ng/mg dry weight for the freeze-thaw, Triton X-100, and SDS treatments, respectively, where *P* < 0.05 compared to the native ADSC sheets (446 ± 38 ng/mg) for all cases. Moreover, the DNA quantification also suggested that more than approximately 95% of the nuclear material was removed by the decellularization processes of Triton X-100 and SDS method (*P* > 0.05). Thus, according to the DNA quantification, H&E staining, and SEM assessment, the Triton X-100 decellularization method was screened to use for further experiments, which can either remove cell components or preserve collagen fiber structure.

### 3.3. Analysis of Retained Cytokines and Mechanical Testing

**Figure 3:**
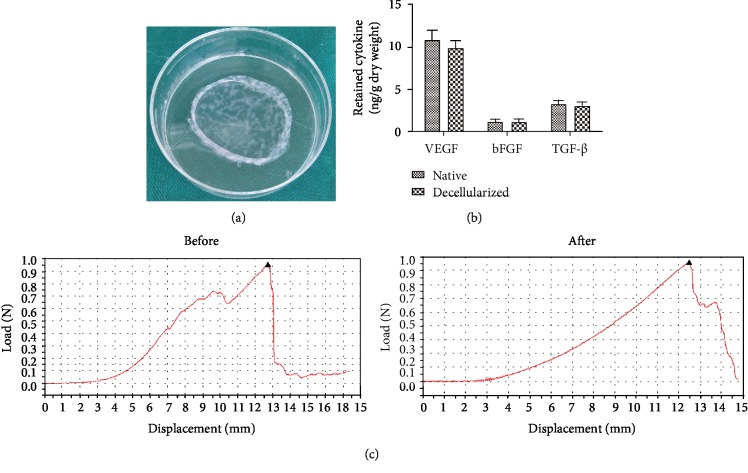
Analysis of retained cytokines and mechanical testing. (a) The gross appearance of decellularized ADSC sheets. (b) ELISA analysis of retained cytokines. The cytokine content, including VEGF, bFGF, and TGF-*β*, has no statistical difference between in decellularized ADSC sheets and natural ADSC sheets (*P* > 0.05). (c) Comparison of load-displacement curve before and after decellularization.

The mean thickness of those decellularized ADSC sheets and ADSC sheets was 67 ± 6.4 *μ*m and 74 ± 8.1 *μ*m, respectively. The decellularized ADSC sheets have a certain mechanical strength and are easy to be handled and transferred (Figure 3(a)). The retention of cytokines was determined by ELISA analysis of protein extractions from the natural and decellularized ADSC sheets (Figure 3(b)). The cytokines were abundant in native ADSC sheets, including significant amounts of VEGF (10.820 ± 1.858 ng/g dry weight), bFGF (1.208 ± 0.388 ng/g dry weight), and TGF-*β* (3.268 ± 0.625 ng/g dry weight). Though the cytokine content decreased slightly during decellularization, high levels of VEGF (9.872 ± 1.363 ng/g dry weight), bFGF (1.165 ± 0.462 ng/g dry weight), and TGF-*β* (3.081 ± 0.585 ng/g dry weight) were also present in the decellularized ADSC sheets and the level of the cytokines VEGF, bFGF, and TGF-*β* in decellularized ADSC sheets was similar to that of the natural ADSC sheets (*P* > 0.05), which could contribute to cell adhesion, migration, proliferation, and differentiation. A load-displacement curve representative of the mechanical behavior of ADSC sheets before and after decellularization is shown in Figure 3(c). There was no significant difference in the mechanical parameters for ADSC sheets before and after decellularization (*P* > 0.05). Detailed parameters are shown in Table 1.

### 3.4. Recellularization Capacity

**Figure 4:**
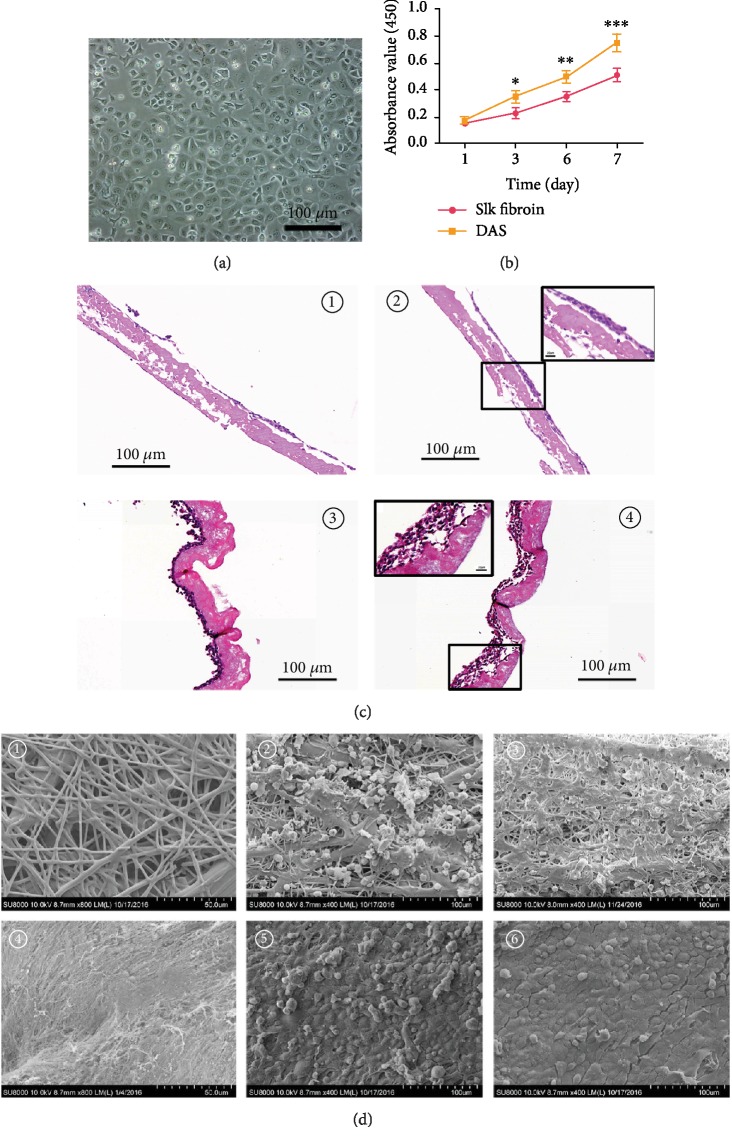
Recellularization capacity of decellularized ADSC sheets in comparison to conventional silk fibroin. (a) Oral mucosal epithelial cell acquisition and culture. (b) Cell proliferation assessment by CCK-8 assay; the decellularized ADSC sheets exhibited a higher proliferative capacity than silk fibroin on days 1, 3, 5, and 7 after cell seeding, and the gap of absorbance value was widening with time; silk fibroin vs. DAS on day 3 after cell seeding (^∗^*P* < 0.05); silk fibroin vs. DAS on day 5 after cell seeding (^∗∗^*P* < 0.05); silk fibroin vs. DAS on day 7 after cell seeding (^∗∗∗^*P* < 0.01). (c) H&E staining observation after cell inoculation: ① silk fibroin recellularization at 3 days of culture, ② silk fibroin recellularization at 7 days of culture—the black box represents a high magnification photograph (400x), ③ decellularized ADSC sheets recellularization at 3 days of culture, and ④ decellularized ADSC sheet recellularization at 7 days of culture—the black box represents a high magnification photograph (400x). (d) SEM observation after cell inoculation: ① morphology of silk fibroin, ② morphology of silk fibroin recellularization at 3 days of culture, ③ morphology of silk fibroin recellularization at 7 days of culture, ④ morphology of decellularized ADSC sheets, ⑤ morphology of decellularized ADSC sheet recellularization at 3 days of culture, and ⑥ morphology of decellularized ADSC sheet recellularization at 7 days of culture. DAS = decellularized ADSC sheets.

To determine the recellularization capacity of decellularized ADSC sheets, oral mucosal epithelial cells (Figure 4(a)) were seeded onto the decellularized ADSC sheets and silk fibroin and examined for cell engraftment and cell growth status. The CCK-8 assay showed that the absorbance value steadily increased with time in two scaffolds, whereas the decellularized ADSC sheets exhibited a higher proliferative capacity than silk fibroin at any time point (Figure 4(b)). The H&E staining result revealed, compared with silk fibroin, oral mucosal epithelial cells proliferated rapidly merging into lines, attached tight to the decellularized ADSC sheets at day 3. In addition, the oral mucosal epithelial cells grew layer by layer and a certain thickness epithelial layer (4-6 layers) formed at day 7 post seeding (Figure 4(c)). The SEM result showed that the cells are tightly connected and only a few necrotic cells were presented on decellularized ADSC sheets at the 3 days of culture and the typical cobblestone-like morphology was displayed on decellularized ADSC sheets at day 7 post seeding, while for silk fibroin recellularization, there are many necrotic cells and partially exposed silk fibroin structure can be seen at 3 days of culture; although cell density increased significantly at 7 days of culture, there are still a few defects and unable to form a cell fusion structure (Figure 4(d)). Thus, the H&E staining and Cell Counting Kit-8 assay confirmed the better recellularization capacity of decellularized ADSC sheets than silk fibroin.

### 3.5. Biocompatibility Test

**Figure 5:**
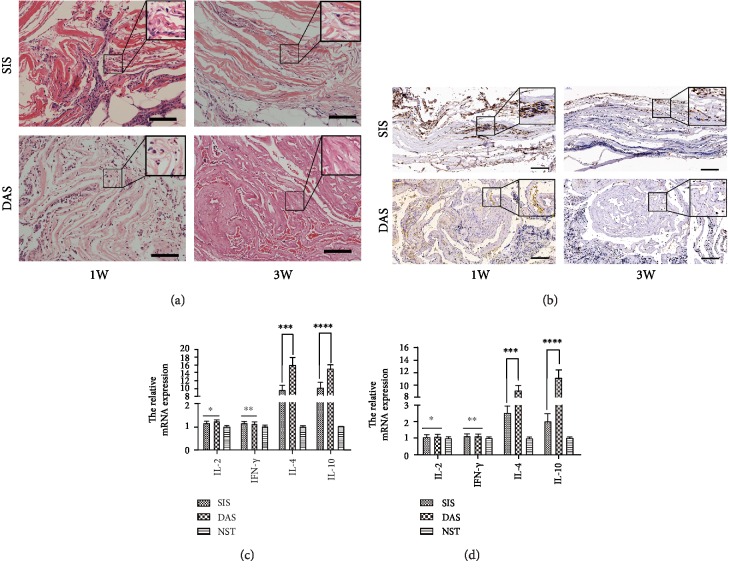
In vivo evaluation of immune rejection and inflammatory response to ECM scaffolds. (a) H&E staining showing host response to decellularized ADSC sheets and xenogenic SIS at 1 week and 3 weeks after implantation. The decellularized ADSC sheets induced much less inflammation than xenogenic SIS during 3 weeks and showed less mononuclear cells and neutrophil granulocyte infiltrating. The black box represents a high magnification photograph (400x) to appreciate the cellularity of implants. Scale bars are of 100 *μ*m. (b) The percentage of CD68+ cells in SIS transplantation was significantly higher than that of the decellularized ADSC sheet transplantation at 1 week and 3 weeks after implantation. The number of inflammatory CD68+ cells in both groups decreased over time, but inflammatory CD68+ cell retrogression is more obvious in the decellularized ADSC sheet transplantation. The black box represents a high magnification photograph of CD68+ cells. Scale bars are of 100 *μ*m. (c) The mRNA expression of multiple cytokines related to immunity and inflammation after 1 week in vivo transplantation; IL-2 comparison of SIS vs. DAS (^∗^*P* > 0.05); IFN-*γ* comparison of SIS vs. DAS (^∗∗^*P* > 0.05); IL-4 comparison of SIS vs. DAS (^∗∗∗^*P* < 0.05); IL-10 comparison of SIS vs. DAS (^∗∗∗∗^*P* < 0.05). (d) The mRNA expression of multiple cytokines related to immunity and inflammation after 3 weeks in vivo transplantation; IL-2 comparison of SIS vs. DAS (^∗^*P* > 0.05); IFN*-γ* comparison of SIS vs. DAS (^∗∗^*P* > 0.05); IL-4 comparison of SIS vs. DAS (^∗∗∗^*P* < 0.01); IL-10 comparison of SIS vs. DAS (^∗∗∗∗^*P* < 0.01). DAS = decellularized ADSC sheets; SIS = small intestinal submucosa; NST = normal subcutaneous tissue. The dog GAPDH was chosen as the reference gene for quantitative real-time PCR.

We assessed the biocompatibility of decellularized ADSC sheets in vivo transplantation for 1 week and 3 weeks; then histological analysis was performed with H&E staining. Both of the decellularized ADSC sheets treated with Triton X-100 and SIS transplantation showed significant inflammatory reaction in the short-term period of 1 week (Figure 5(a)). It was found that inflammatory cells, containing mononuclear cells and neutrophil granulocyte, were infiltrating mainly in the transplantation site. After 3 weeks of implantation, the number of infiltrated inflammatory cell significantly decreased as time goes by, especially for decellularized ADSC sheets (Figure 5(a)). CD68 staining was used to evaluate monocytes and macrophage infiltration in the transplantation site (Figure 5(b)). The detection of Th-related cytokines from the transplantation site is valuable to understand the state of local immune response. The Th1/Th2 cytokine mRNA expression in the transplantation site was further determined by qPCR, as shown in Figures 5(c) and 5(d). On either 1 week or 3 weeks after transplantation in the normal subcutaneous tissue, the mRNA expression levels for IL-2 and IFN-*γ* did not show a statistical difference between decellularized ADSC sheets and xenogenic SIS (*P* > 0.05). Furthermore, both groups were associated with a significant rise in the mRNA levels of IL-4 and IL-10 than the normal subcutaneous tissue at 1 week after transplantation; however, the mRNA levels of IL-4 and IL-10 of decellularized ADSC sheets dropped rapidly and were apparently higher than those of xenogenic SIS at 3 weeks after transplantation (*P* < 0.05).

## 4. Discussion

Decellularized ECM-based biomaterials have shown significant application prospect as surgical implants and scaffolds for tissue engineering. In our study, the optimal decellularized procedure was explored to prepare an ADSC sheet-derived ECM bioscaffold, which retains intact ECM ultrastructure and abundant cytokines, and the cells seeded on such biomimetic scaffolds could potentially produce functional tissue-engineered grafts for regenerative medicine.

The classical decellularization methods include physical, chemical, and biological treatments and their combinations, but there is no single technique that is appropriate for various tissues and organs. The most effective agents for decellularization will depend upon multifactors, including the tissue's cellularity, lipid content, density, and thickness [20]. An ideal decellularization approach can remove all the cellular components and retain the integrity of the ECM and microstructure. In this study, we firstly selected the optimal decellularization protocol to prepare the decellularized ADSC sheets. The results of DNA quantification and H&E staining are used as the quantitative markers of cellular remnants [7]: (1) DNA quantification less than 50 ng/mg ECM dry weight and (2) invisible nuclear material stained with H&E. Repeated freeze-thaw induces the formation of intracellular ice crystals and may disrupt the cell membrane and cellular structure, leading to tissue disintegration and cell death. Freeze-thaw processing has been proved to produce minor disruptions of the ECM ultrastructure, preserve elastin amount, and retain the mechanical properties [24]. However, these freeze-thaw treatments are generally insufficient to achieve complete decellularization, and the process must be followed by other decellularization processes. In the present study, the resulting DNA content remained large in the decellularized ADSC sheets, reaching up to 48.5% ± 4.3% for freeze-thaw treatments (Figure 2).

Ionic detergents are efficient for solubilizing nuclear and cytoplasmic cellular membranes but tend to denature proteins by disrupting protein-protein interactions [25]. SDS is the most commonly used ionic detergent for removing cellular remnants. Compared with Triton X-100, SDS is typically more effective in cell removal but is associated with greater disruption of the ECM ultrastructure [2]. For the pericardium, it has been shown that decellularization with 1% SDS causes irreversible swelling and disruption of collagen fiber and decrease in tensile strength compared to native tissues [26]. As a thin ECM sheet, the thickness and toughness of ADSC sheets are less than those of the native tissue, such as pericardium, and we reduced the concentration of SDS to 0.25%. Though the DNA quantification and H&E staining indicated the high clearance rate of the cellular components (Figure 2), however, the elastic fiber distribution and collagen network were disrupted in the decellularized ADSC sheets after SDS treatment (Figure 2). Nonionic detergents break lipid-protein and lipid-lipid interactions but leave intact protein-protein interactions and maintain the inherent structures [27]. Triton X-100 is the most widely applied nonionic detergent for decellularization procedures and is more suitable to eliminate cells from thin tissues than from thick and complex tissues [28]. The collagen content of a decellularized skeletal muscle in the Triton X-100 groups is nearly fully preserved compared to an approximately 27% to 31% reduction in the trypsin-Triton X-100-SDS group and the trypsin-SDS group [29]. The clearance ratio of DNA and cell components in Triton X-100 sample was similar to that of the SDS sample, but it maintained the ECM microenvironments and had no effect on the collagen fiber structure (Figure 2); thus, the Triton X-100 was screened to be used for ADSC sheet decellularization in our experiments.

ADSCs can secrete a broad array of growth factors and cytokines that modulate the inflammatory response, promote angiogenesis, and recruit endogenous stem cells, which may contribute to co-ordinate in situ tissue regeneration [30]. Furthermore, stem cells secrete a large amount of endogenous ECM, which provides a native cell microenvironment in strengthening cell adhesion, proliferation, differentiation, migration, and tissue morphogenesis. Through binding to specific ECM molecules, various cytokines, growth factors, and chemokines are secreted and deposited within ECMs [31]. The preserved ultrastructure and composition of the ECM also help growth factor storage and release. Cytokine production is relatively slow, and the mature cytokine combined with ECMs accounts for the majority in the ADSC sheets. Chun et al. [32] reported that the bladder acellular matrix preserved abundant endogenous growth factors after the decellularization processing, including bFGF, VEGF, and TGF-*β*, and that it is similar to our decellularized ADSC sheets (Figure 3(b)). Meanwhile, ECM can provide an ideal microenvironment to accelerate circulation and exchange of oxygen and nutrient between cells and external environment. As a natural scaffold material, previous studies showed that silk fibroin could be shaped into a sheet or film to support the formation of epithelium and displayed significant advantages, including biocompatibility, moderate mechanical, properties and low cost [33–35]. However, like any other nonautologous biomaterials, silk fibroin can induce some adverse immunological events for its nonmammalian origin. Thus, we compared the cell-seeding potential with silk fibroin in vitro to avoid the immunological rejection. Different from silk fibroin, the decellularized ADSC sheets as a scaffold provide a “native” biological environment for cell attachment and preserve a considerable amount of growth factors to promote cell proliferation, and deservedly, the use of thin ECM sheets allows much easier recellularization than synthetic scaffold, and the result of experimental studies is consistent with the theory analysis during 7 days after oral mucosal epithelial cell inoculation. Oral mucosal epithelial cells seeded on decellularized ADSC sheets showed superior proliferation ability compared to that on silk fibroin (Figure 4). Extracellular matrix has a three-dimensional structure to promote cell adhesion and differentiation and also provides a certain mechanical strength for tissue regeneration. Thus, it can be used as a satisfactory repair material for soft tissue regeneration.

SIS is rich in collagen, glycosaminoglycans, and growth factors, which has been used as a biomaterial scaffold for bone, ligament, skin defects, blood vessel, abdominal body wall, urethral stricture, and dura in a clinical and preclinical study. In addition, many studies showed SIS has little risk of rejection because it is acellular. Thus, we compared the histocompatibility and immune rejection with SIS in vivo. Nucleic DNA and cell membrane epitopes are responsible for adverse inflammatory and immune responses in xenogenic or allogeneic organ transplants. The decellularized process can cause the loss of cell membrane receptor, which greatly reduces the cell antigenicity. However, the cell membrane and nuclear component is hard to be completely eliminated and the host immune response will be activated by residual cell components and xenogenic protein. In addition, the heterogeneous components of acellular matrix still have certain immunogenicity, which can produce immune rejection and even cause death of patients after transplantation [36]. In the study, the inflammatory response was examined histologically after 1 week and 3 weeks in vivo transplantation, and the result indicated acute inflammatory cell infiltration was induced by both the decellularized ADSC sheets and xenogenic SIS at the early stage (1 week) and mainly concentrated at the interface of organization and autografts, which could be a nonspecific postoperative inflammatory reaction (Figure 5(a)). However, H&E staining is not sufficient to characterize inflammatory cell accumulation in the samples. CD68 is a 110 KD transmembrane glycoprotein and highly expressed by monocytes and tissue macrophages. Compared to decellularized ADSC sheet implantation, the SIS implantation expressed significantly more CD68 at different time points, which confirmed SIS implantation developed a more severe inflammatory response (Figure 5(b)). Mice implanted with xenogenic SIS produced a SIS-specific antibody response, CD3^+^ T cell infiltration, and Th2-like immune response [37]. Th2 response was associated with the donor's body accepting the xenogeneic proteins, tissue, or organs [38]. The inflammatory reaction disappeared, and monocytes and macrophages significantly decreased in the decellularized ADSC sheets at 3 weeks of transplantation, while the inflammation still remains in the transplantation site of xenogenic SIS, which demonstrated the decellularized ADSC sheets possess excellent biocompatibility. Similarly, the mRNA expression of Th-related cytokine obtained by qPCR was in accordance with the result of histological analysis. Th1 cells have been proved to play a major role in antitumour immunity and stimulation of cell-mediated responses. Proinflammatory cytokines such as TNF-*α* and IFN-*γ* are known to stimulate Th1 cells. In contrast, Th2 cells are known to act as the helper cells that influence B-cell development and produce anti-inflammatory cytokines such as IL-4 and IL-10. The Th1-related cytokine (IL-2 and INF-*γ*) mRNA expression showed no significant difference in both groups, and the expression quantity of them is similar to normal subcutaneous tissue, which indicated both xenogenic SIS and decellularized ADSC sheets did not cause T cell-mediated immune rejection. However, Th2-related cytokine (IL-4 and IL-10) mRNA expression was significantly higher in the xenogenic SIS than in the decellularized ADSC sheets, which showed that xenogenic SIS was immunogenic and immune response was a humoral immunity process guided by a specific xenogeneic protein and mediated by Th2 lymphocyte after xenogeneic transplantation. In addition, the high expression of IL-4 and IL-10 promotes macrophage polarization to M2 anti-inflammatory phenotype which could be beneficial for mitigation of local inflammatory response to implants, promoting angiogenesis, tissue remodeling, and repair [39]. In a further analysis, compared to the normal subcutaneous tissue, high gene expression of IL-4 and IL-10 was found in both groups at 1 week and 3 weeks after transplantation, which indicated both SIS and decellularized ADSC sheets can induce the anti-inflammation effect. However, the mRNA levels of IL-4 and IL-10 of decellularized ADSC sheets are apparently higher than those of xenogenic SIS at 3 weeks after transplantation, which showed the decellularized ADSC sheets provided the more powerful and long-lasting anti-inflammatory effect. Previous research suggested mesenchymal stem cells promote the preferential shift of the macrophage phenotype from M1 to M2 and contribute to tissue repair [40]; thus, we speculate that the anti-inflammatory effect of decellularized ADSC sheets was related to immune-modulating characteristics of ADSC.

In this experiment, we developed an acellular matrix using ADSC sheets with Triton X-100 decellularization and preliminarily evaluated its characteristic of cellular compatibility and immunoreactivity. However, the advantages and disadvantages of a biomaterial need to be evaluated in many ways and some limitations should be considered in this study. Firstly, we just only detected a few retained cytokine levels; a wider cytokine analysis should be performed in a future experiment. In addition, biomaterial-mediated immunity is a complex process in vivo. Our study indicated the decellularized ADSC sheets possessed better biocompatibility than SIS, but the underlying mechanisms for decellularized ADSC sheets inducing less inflammation remain unknown and it needs further investigation. A further study will be needed to explore the host immune mechanism after decellularized ADSC sheet transplantation and provide more detailed information for the suitability of these biological scaffolds with or without cell seeding for repairing and reconstruction of the damaged soft tissues and organs *in vivo*.

## 5. Conclusion

We established a novel bioscaffold through the combined use of cell sheet technology and Triton X-100 decellularization protocols. The ADSC sheet-derived ECM scaffolds preserved the three-dimensional architecture of ECM, retained the mechanical strength, exhibited good recellularization potential and less inflammation, and possess good application prospect for autogenous and/or allogenic soft tissue augmentation and reconstruction.

## Figures and Tables

**Table 1:** The mechanical properties of the decellularized ADSC sheets and ADSC sheets.

	Elasticity modulus (MPa)	Maximum load (*N*)	Maximum tensile displacement (mm)
ADSC sheets	0.142 ± 0.029	0.931 ± 0.118	12.833 ± 1.583
Decellularized ADSC sheets	0.129 ± 0.021	0.893 ± 0.107	12.384 ± 1.471

## Data Availability

The data used to support the findings of this study are included within the article.
